# Journal of Molecular Psychiatry

**DOI:** 10.1186/2049-9256-1-1

**Published:** 2013-04-23

**Authors:** Johannes Thome

**Affiliations:** Clinic and Policlinic for Psychiatry and Psychotherapy, University of Rostock, Gehlsheimerstr. 20, Rostock, 18147 Germany

It is my pleasure to welcome you to the new Journal of Molecular Psychiatry – JMP! Molecular Psychiatry is a rapidly expanding field and there are high hopes that its findings will improve the condition of patients suffering from psychiatric disorders in terms of diagnosis, prognosis and therapy. There is only a limited number of journals specialising in this exciting area, and we believe that a modern internet-based electronic journal fully dedicated to the open-access principle with a thorough and efficient peer-review process and without space restrictions, will serve well the communication and publication needs of the scientific community. All articles published in *JMP* will be open access, therefore complying with the open access policies of funders including the Howard Hughes Medical Institute [1], NIH [2], and Wellcome Trust [3].

We are proud that one of the first publications in *JMP* is authored by Eric Nestler [4], who - together with Ron Duman – coined the term “Molecular Psychiatry” in the 1980s. Considered by many as the “Father of Molecular Psychiatry” and an eminent researcher, he critically reviews the historical development this discipline has undertaken during the last 30 years. He points out chances and opportunities, but also mentions mishaps and disappointments.

While the term “molecular psychiatry” is widely used, surprisingly little has been published about its meaning and conceptualisation. Many seem to use the term as a more contemporary synonym for “biological psychiatry”. However, “biological psychiatry” usually represents a quite reductionist view of mental health and psychiatric disorders as phenomena which can and should exclusively be understood and explained in a mechanistic way based on neuroscience and pathophysiology. Thus, human behaviour is interpreted as the result or simple metaphenomenon of processes occurring on the cellular, genetic and molecular level. Unfortunately, this does not reflect the reality of clinical practice and the humanistic roots of psychiatry which are equally important as its biological aspects. It is one of the great achievements of molecular psychiatry to unravel the oversimplification of many theories in biological psychiatry and to open our eyes to fundamental processes such as gene-environment interaction which enable us to integrate even psychotherapeutic and social concepts of mental health into a new holistic and integrative psychiatry. Therefore, we are planning to not only publish high-quality research based on molecular neuroscience, but also reviews of mental health research in the context of Molecular Psychiatry.

I am very grateful to the editorial board [5] consisting of an international panel of eminent scientists and psychiatrists for its support. I am also thankful to the publishers BioMed Central and Springer Vienna who helped to realise this project, especially Mrs Schilgerius and Ms Ridgway who are handling with their teams all the logistic and infrastructural procedures needed for the successful production of the articles. We all have worked hard during the last months to launch *JMP* and are proud of what we have achieved. The following publications prove that *JMP* already is a great success.
